# Perspective on Particulate Matter: From Biomass Burning to the Health Crisis in Mainland Southeast Asia

**DOI:** 10.3390/toxics11070553

**Published:** 2023-06-23

**Authors:** Teerachai Amnuaylojaroen, Nichapa Parasin

**Affiliations:** 1 School of Energy and Environment, University of Phayao, Phayao 56000, Thailand; 2 Atmospheric Pollution and Climate Research Unit, School of Energy and Environment, University of Phayao, Phayao 56000, Thailand; 3 School of Allied Health Science, University of Phayao, Phayao 56000, Thailand; nichapa.pa@up.ac.th

**Keywords:** air quality, Southeast Asia, PM_2.5_, public health, particulate matter

## Abstract

Air pollution, notably particulate matter pollution, has become a serious concern in Southeast Asia in recent decades. The combustion of biomass has been recognized to considerably increase air pollution problems from particulate matter in this region. Consequently, its effect on people in this area is significant. This article presents a synthesis of several datasets obtained from satellites, global emissions, global reanalysis, and the global burden of disease (GBD) to highlight the air quality issue and emphasize the health crisis in mainland Southeast Asia. We found that the death rates of people have increased significantly along with the rise of hotspots in mainland Southeast Asia over the last two decades (2000–2019). In comparison, most countries saw a considerable increase in the predicted fatality rates associated with chronic respiratory illnesses during those two decades. Several reports highlight the continued prevalence of chronic respiratory diseases likely related to poor air quality in Southeast Asia.

## 1. Introduction

The escalation of air pollution caused by particulate matter is a noteworthy factor contributing to the deterioration of air quality, which is increasingly becoming an urgent concern in various regions internationally [1]. Particulate matter with a diameter of less than 2.5 mm is commonly referred to as fine particulate matter or PM_2.5_. The particles possess the ability to penetrate deeply into the lungs and bloodstream owing to their microscopic size, thereby leading to a diverse range of detrimental impacts on human health [1]. The study conducted by Pope et al. [2] in the United States found that prolonged exposure to PM_2.5_ pollution, even at concentrations lower than the current air quality regulations set by the federal government, was associated with an increased risk of mortality due to lung cancer. A recent study conducted by Liu et al. [3] has established a correlation between inhaling PM_2.5_ pollution and increased susceptibility to stroke in China. In addition, Carey et al. [4] found that prolonged exposure to PM_2.5_ in the United Kingdom was associated with an increased likelihood of mortality resulting from cardiovascular disease. At the same time, Atkinson et al. [5] found a positive correlation between short-term exposure to PM_10_ and an increased probability of hospitalization due to cardiovascular disease in London.

In Southeast Asia, the issue of air pollution caused by biomass burning is a major concern for both public health and the environment, with significant consequences, as noted by Amnuaylojaroen et al. [6,7]. Biomass burning refers to combusting organic matter, such as trees and crop residues, for agricultural purposes. In mainland Southeast Asia, biomass burning is a common practice. It is extensively used in rural areas for a variety of functions, including land clearance, cooking, and heating [8]. Keywood et al. [9] proposed that the release of fumes and particulate matter resulting from biomass burning substantially impacts air quality, human health, and ecology. Several studies have shown that biomass combustion is a significant contributor to increasing levels of fine particulate matter (PM_2.5_) in mainland Southeast Asia, resulting in air pollution. Also, PM_2.5_ concentrations in mainland Southeast Asia tend to increase in the future [10]. During the dry season, biomass-burning emissions were shown to be responsible for as much as 70% of PM_2.5_ concentrations. Furthermore, the emissions from biomass burning have major effects on the environment, including climatic changes and ecological deterioration [11]. In comparison, Nhung et al. [12] discovered that inhaling PM_2.5_ from biomass burning relates to increased sensitivity to respiratory and cardiovascular disorders in rural Vietnamese households. Moreover, exposure to PM_2.5_ is associated with a 69% and 29% increase in the likelihood of hospitalization for respiratory and cardiovascular disorders, respectively.

Air pollution generally has detrimental effects on health because it has been identified as a primary cause of death from stroke, heart disease, and respiratory diseases [13]. Certain demographic groups, including children, the elderly, and individuals with pre-existing health conditions, have been found to be particularly vulnerable to the negative effects of PM_2.5_ pollution [7,14,15]. This type of pollution has the potential to worsen symptoms and result in serious adverse health effects. Previous studies have underscored the significant impact of PM_2.5_ pollution on the health and welfare of individuals residing in mainland Southeast Asia, as noted by Sun et al. [16]. Several research studies have been carried out to investigate the impact of fine particulate matter on human health in mainland Southeast Asia. Chankaew et al. [17] conducted a study in Thailand and found that there appears to be a correlation between the occurrence of asthma exacerbations and the high PM_2.5_ season. According to the findings of Nhung et al. [18], research conducted in Vietnam, there exists a positive association between exposure to PM_2.5_ and the probability of developing cardiovascular disease. The conditions encompass an increased vulnerability to cardiovascular and respiratory disorders, cognitive decline, adverse pregnancy outcomes, elevated cancer risk, and mental health complexities. The studies mentioned above emphasize the importance of prioritizing the reduction of air pollution as a public health concern in this region. This involves implementing regulations that aim to reduce emissions and improve air quality.

The investigation pertaining to the issue of air quality and its impact on human health in Southeast Asia is not new. Despite the existence of various studies on the correlation between air quality and its effects on human health in Southeast Asia, no tangible measures have been taken to address this issue in the region. Understanding the fundamental factors that contribute to the issue of air quality and exploring feasible solutions to mitigate its impact on human health and the environment is the most important. This article aims to provide a perspective on the issue of air quality concerns related to particulate matter over the last decade. Additionally, it highlights the critical impact of air quality issues on human health in this region. The current undertaking is of substantial importance and requires a significant time to achieve a consensus satisfactory to all stakeholders, including governmental authorities, local authorities, tribal communities residing in the hills, and the general public. From our perspective, it is crucial to underscore the need for implementing measures that are targeted at mitigating the issue of particulate matter (PM) pollution in mainland Southeast Asia. This involves the implementation of strategies aimed at reducing emissions arising from the combustion of biomass and its related sources. Moreover, it is crucial to enhance the dissemination of information and education to the general public regarding the health implications of air pollution.

## 2. Data Used

**Figure 1:**
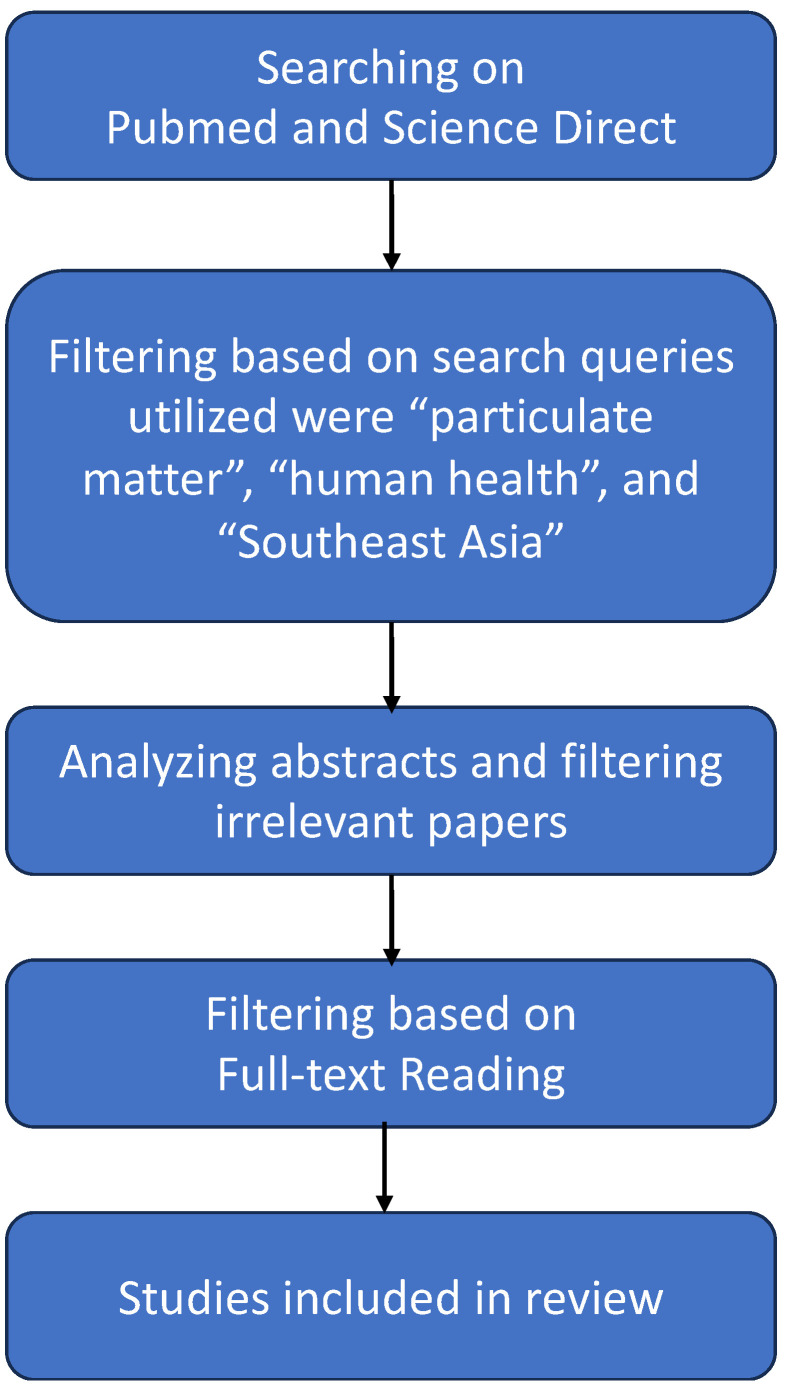
The flowchart of the literature search.

In order to elucidate the viewpoint regarding air quality issues, from particulate matter to the health crisis in mainland Southeast Asia, we conducted a literature search utilizing two databases, namely PubMed and Science Direct (Figure 1). The title, abstract, and keyword of a given string were thoroughly scrutinized for all search terms. The search queries utilized were “particulate matter”, “human health”, and “Southeast Asia”. The selection of the pertinent literature from the past decade was based on the sufficiency of data quantity and the reliability of data precision.

The issue of air quality in mainland Southeast Asia was identified through the utilization of multiple datasets, which included emission data from both anthropogenic and biomass-burning sources obtained from the Emissions Database for Global Atmospheric Research version 6 (EDGARv6) [19] and the Global Fire Emissions Database version 3 (GFED3) [20], respectively. The Modern-Era Retrospective Analysis for Research and Applications, Version 2 (MERRA-2), was employed to analyze the spatial distribution of PM_2.5_ and aerosol optical depth (AOD). NASA’s Global Modeling and Assimilation Office has developed MERRA-2, the most recent atmospheric and aerosol reanalysis product [21]. The MERRA-2 AOD present in the model results from combining the aerosol mixing ratio, extinction coefficient, and relative humidity. The aerosol optical depth (AOD) is represented as the aggregate of the AOD values of five distinct aerosol types, namely black carbon (BC), organic carbon (OC), dust, sea salt, and sulfate. In contrast, the determination of the concentration of fine particulate matter (PM_2.5_) on the surface involves the summation of distinct aerosol components, namely organic carbon, black carbon, sulfate, sea salt, and dust, as per the methodology proposed by Buchard et al. [22]. Furthermore, this concentration is transformed from the original MERRA-2 model grid. The hotspot data obtained from VIIR measurements were utilized to ascertain the number of fire sources present in Southeast Asia. The VIIRS instrument conducts measurements across 22 spectral bands, encompassing a range of wavelengths spanning from 0.41 to 12.0 m. The satellite comprises twenty-one bands, of which sixteen are classified as moderate resolution bands, possessing a nadir spatial resolution of 750 m. The remaining five bands are categorized as imaging (I) bands, with a 375 m nadir spatial resolution. The VIIRS possesses a distinctive day/night band (DNB) within the reflective solar spectrum (0.5–0.9 m) that operates at three distinct gain stages. This feature facilitates an extensive dynamic range and permits the acquisition of data during both diurnal and nocturnal orbits. The VIIRS Remote Sensing Bands (RSB) encompass a range of spectral bands, specifically M1–11 and I1–3, while the Thermal Emissive Bands (TEB) comprise bands M12–16 and I4–5. The VIIRS spectral bands are distributed across three focal plane arrays (FPAs), which are designated as VIS/NIR, S/MWIR, and LWIR. According to Xiong and Butler [23], the S/MWIR and LWIR FPAs are maintained at a temperature of 80 K during nominal operations.

The health crisis in Southeast Asia was revealed through health data, specifically the number of deaths, disability-adjusted life years, and years of life lost from chronic respiratory disease obtained from the Global Burden of Disease. A fixed-effect model was employed to assess the correlation between chronic respiratory disease (CRD) mortality and various factors such as gross domestic product (GDP) per capita, average years of schooling, urbanization, and pollutant emissions.

## 3. Air Quality in Mainland Southeast Asia

**Figure 2:**
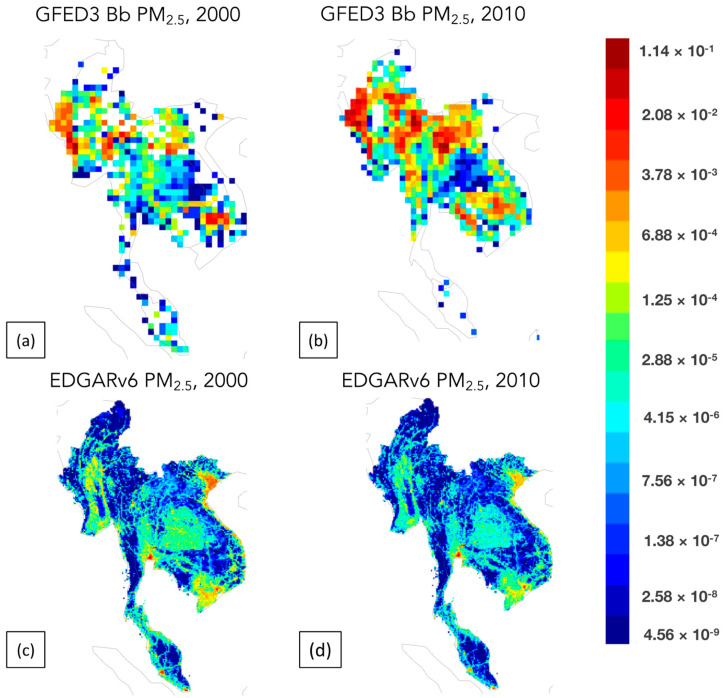
Spatial distribution of PM_2.5_ from biomass burning emission from GFED in (**a**) 2000 and (**b**) 2010, and from anthropogenic emission from EDGARv6 in (**c**) 2000 and (**d**) 2010. (https://eccad.aeris-data.fr, accessed on 16 June 2023).

Ramanathan et al. [24] have extensively discussed the topic of air pollution in Southeast Asia, particularly over the continent, for several decades. PM_2.5_ is the most important air pollutant contributing to air quality in this region [25]. The PM_2.5_ issue in this region, similar to other global regions, can be broadly categorized into two types: anthropogenic sources, which comprise emissions from industrial activities and transportation, and biomass burning emissions, which involve uncontrolled combustion of agricultural waste and forest fires. Amnuaylojaroen et al. [25] and Duc et al. [26] have reported the observation of pollutant peak periods, particularly with respect to particulate matter, during the dry season, specifically from February to April. According to Amnuaylojaroen et al. [8], emissions from biomass burning, notably those resulting from agricultural and forest fires, have a considerable influence on PM_2.5_ pollution in the region. In addition, particulate matter (PM) released through biomass combustion has a significant impact on the atmosphere, both directly and indirectly, by altering the level of atmospheric radiation [27]. According to Bond et al. [28], biomass burning is a substantial source of fine particulate matter (PM_2.5_), accounting for 41% and 74% of the world’s total primary black carbon and organic carbon emissions, respectively [29,30]. The emission data plot of PM_2.5_ is presented in Figure 2, which encompasses both anthropogenic sources from the Emissions Database for Global Atmospheric Research version 6 (EDGARv6) [19] and natural sources from the Global Fire Emissions Database version 3 (GFED3) [20]. The EDGAR and GFED databases provide comprehensive information on emissions, including national amounts and grid maps at a resolution of 0.1 and 0.25 degrees for EDGAR and GFED, respectively, on a global scale. The data are available for various timeframes, from yearly to monthly and even hourly intervals. The data presented illustrate the annual distribution of emissions resulting from biomass burning in mainland Southeast Asia during the years 2000 and 2010. The research findings indicate that there is a significant rise in biomass burning emissions in the mainland Southeast Asia area in comparison to anthropogenic emissions. Compared to the year 2000, there was a significant rise in biomass burning emissions up to 0.0208 Tg, with a specific focus on Myanmar, Laos, and the northern part of Thailand.

**Figure 3:**
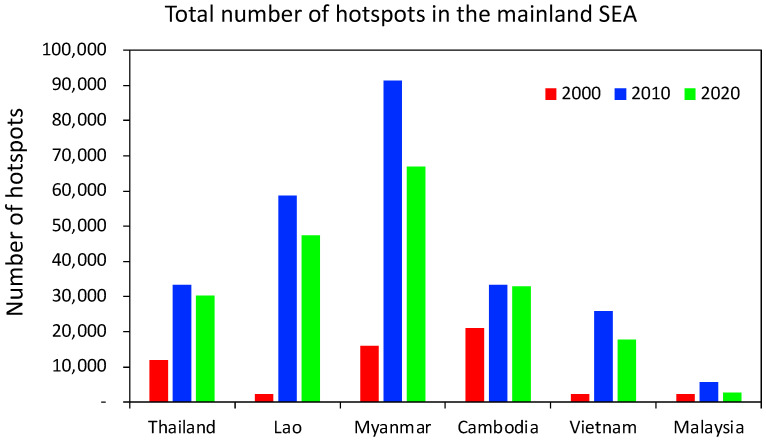
Total number of hotspots of countries in mainland Southeast Asia in 2000, 2010, and 2020. (https://firms.modaps.eosdis.nasa.gov/map, accessed on 16 June 2023).

As stated above, biomass burning is the major source of PM, notably PM_2.5_, that contributes to air quality problems in this region. Biomass burning in Southeast Asia is substantially related to fire emissions [31]. In recent decades, there has been a significant rise in biomass burning caused by fires in multiple countries across Southeast Asia [32]. The data from the VIIRS Moderate Imaging Spectroradiometer (MODIS) [33] provide information on the number of fire hotspot locations in various countries, including Thailand, Laos, Myanmar, Cambodia, Vietnam, and Malaysia, for the years 2000, 2010, and 2020 (Figure 3). Based on data obtained in the year 2000, it was observed that Cambodia had the highest number of hotspots, which amounted to 21,077, while Vietnam had the lowest number of hotspots, with a count of 2293. During the subsequent two decades, there was a notable variation in the number of hotspots identified in multiple countries. Myanmar experienced a significant increase in hotspots, with a surge from 15,992 in 2000 to 91,388 in 2010, indicating a remarkable growth of 471%. Nonetheless, there was a notable reduction in the rate of expansion of the aforementioned phenomenon during the subsequent decade. As of the year 2020, the number of hotspots had solely experienced an increase to a total of 66,989. The number of hotspots in Laos experienced a substantial escalation, demonstrating a noteworthy increase of 2383% from 2363 in the year 2000 to 58,684 in the year 2010. The area being analyzed experienced a significant deceleration in the rate of expansion of hotspots over the subsequent decade, similar to that observed in Myanmar. There was a slight increase in the number of hotspots, reaching a total of 47,331 by 2020. Through a period of two decades, Thailand demonstrated a relatively consistent number of hotspots, experiencing a slight decrease from 33,368 in the year 2010 to 30,234 in the year 2020. The number of hotspots in Vietnam endured a significant increase from 2293 in 2000 to 17,821 in 2020. Malaysia demonstrated the lowest count of hotspots in contrast to the remaining five countries during a period of two decades. Although biomass burning is relatively associated with fire emissions [25], meteorological factors, i.e., precipitation, also play a key role in controlling air quality problems in Southeast Asia. Vadrevu et al. [34] revealed the association between fires and precipitation in Southeast Asian countries, finding that precipitation had a high negative correlation with fire numbers. Furthermore, recent research indicates that the majority of the fires in this region are caused by humans. Forests are set on fire for a variety of reasons, including inducing the growth of new grass for grazing, clearing the land for farming, such as cutting and igniting, obtaining minor forest goods such as honey, palatable, and foliage, hunting wild animals, and residue from crops burning in agricultural areas [35,36,37,38].

**Figure 4:**
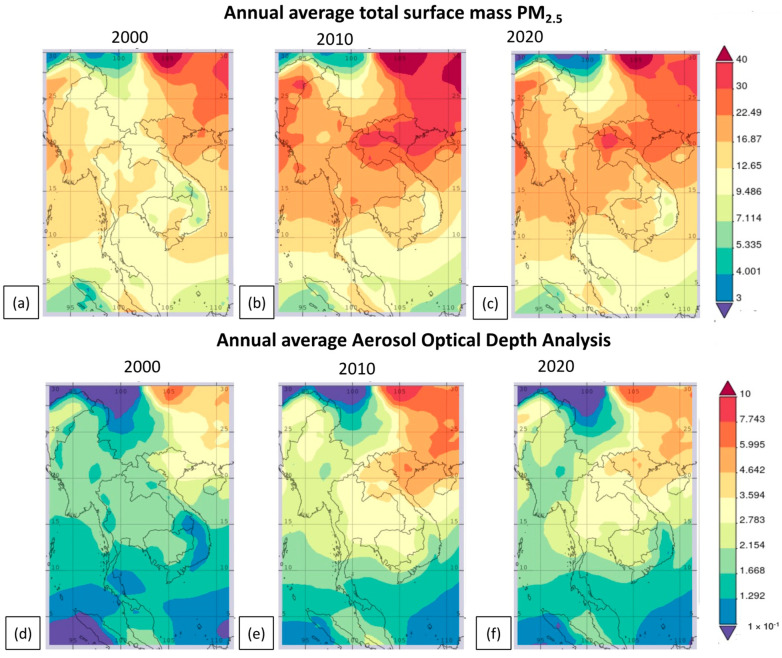
Spatial distribution of surface mass PM_2.5_ in (**a**) 2000, (**b**) 2010, and (**c**) 2020, and aerosol optical depth in (**d**) 2000, (**e**) 2010, and (**f**) 2020 in mainland Southeast Asia. (https://giovanni.gsfc.nasa.gov/giovanni, accessed on 16 June 2023).

The spatial distribution of surface mass PM_2.5_ and aerosol optical depth in mainland Southeast Asia for the years 2000, 2010, and 2020, as obtained from MERRA [39], is depicted in Figure 4. According to the MERRA reanalysis data, levels of PM_2.5_, ranging from 16 to 22 µg/m^3^, were detected in the year 2000 in many countries, such as Thailand, Myanmar, and Laos. The years of 2010 and 2020 witnessed a significant rise in PM_2.5_ levels, which ranged from 22 to 40 µg/m^3^. A reduction in the PM_2.5_ concentration was observed in several regions of Myanmar, Thailand, and Laos in 2020. The spatial distribution of Aerosol Optical Depth (AOD) showed a similarity to the distribution of PM_2.5_ concentration, as illustrated in Figure 4d–f. The AOD demonstrated an increasing pattern in various countries, such as Myanmar, Laos, and Thailand, between 2010 and 2020, as opposed to 2000. The levels of PM_2.5_ concentration in this region were attributed to the increase in hotspots and biomass-burning emissions. The distribution of air quality, especially during the dry season, is dominated by the Asian winter monsoon [25]. From November to March, the Asian winter monsoon circulates air from the Asian continent to the ocean. In March, the winds reached the northern region of Thailand via two primary paths. The primary channel is distinguished by winds traveling from eastern Asia into Laos and the northern part of Thailand, while the additional channel is distinguished by winds moving from Myanmar and entering the northern part of Thailand from the northwest. These circulation patterns transport and distribute trace gases and aerosols released by biomass burning in this region [25].

As previously stated, there has been a discernible trend of increasing concern regarding the issue of air quality degradation caused by particulate matter over the last few decades. Meanwhile, there is a tendency for an increased risk of this issue in the future. Based on recent reports, there is a projected exacerbation of air quality issues related to particulate matter in Southeast Asia. For example, Nguyen et al. [40] conducted a study utilizing a numerical model to analyze the effects of climate and emission changes on mixing. On average, it was projected that PM_2.5_ concentrations would increase between 10 and 21% and 20 and 28% during the dry and wet seasons, respectively, across four specified countries under the RCP4.5 and RCP8.5 scenarios. The findings of the simulation suggest that the fluctuation in PM_2.5_ concentrations is primarily influenced by the emission trend, with climate change also exerting a significant impact. Amnuaylojaroen et al. [10] reported that alterations in climatic parameters and emissions have led to an increase in PM_2.5_ concentrations across northern Peninsular Southeast Asia, ranging from (+1) to (+35) μg/m^3^ during the dry season. Lee et al. [41] have reported that the elevated concentration of PM_2.5_ in Southeast Asia is not solely attributed to biomass burning emissions but also to anthropogenic emissions from sectors such as ship emissions, coal consumption, and biofuel consumption, which are projected to contribute to a 26% and 8.6% increase in PM_2.5_ concentration in the region, respectively.

## 4. Health Effect of Poor Air Quality

**Figure 5:**
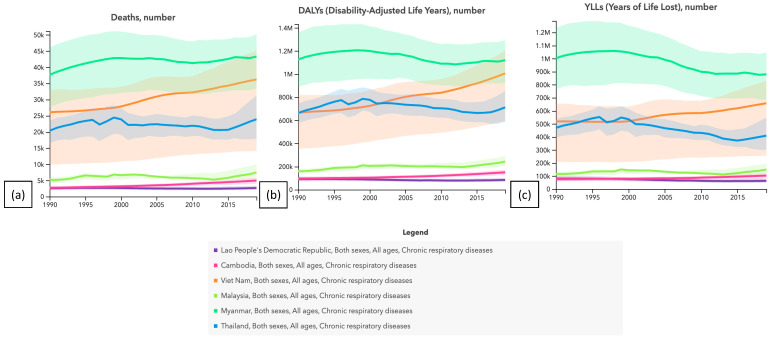
Number of (**a**) deaths, (**b**) disability-adjusted life years, and (**c**) years of life lost in mainland Southeast Asia from chronic respiratory disease during 1990–2019. (https://www.healthdata.org/gbd, accessed on 16 June 2023).

The deteriorating air quality in Southeast Asia over the past few decades has had a direct impact on the health of individuals residing in this region [6]. According to the Global Burden of Disease Study (GBD) 2019 [42], chronic respiratory diseases were responsible for a significant number of deaths in mainland Southeast Asia between 1990 and 2019, as illustrated in Figure 5. The present study provides an assessment of mortality rates linked to chronic respiratory illnesses, such as chronic obstructive pulmonary disease, asthma, and other respiratory diseases, in mainland Southeast Asia, spanning the period from 1990 to 2019. There was an increase in the number of deaths attributed to chronic respiratory diseases in Cambodia from 12,756 in 1990 to 14,413 in 2019. Likewise, it can be observed that in Laos, there has been a rise in the mortality rate from 2096 in 1990 to 3179 in 2019. The mortality rate in Malaysia has exhibited an upward trend, with a rise from 6565 in 1990 to 7754 in 2019. The mortality rate in Myanmar has exhibited an upward trend, with fatalities rising from 38,962 in 1990 to 59,782 in 2019. The mortality rate in the Philippines has shown an upward trend, with a rise from 35,858 in 1990 to 51,186 in 2019. Between 1990 and 2019, there was an increase in the number of deaths in Thailand from 29,630 to 36,750, and in Vietnam, the number of deaths rose from 36,698 to 48,568 during the same period. The report by GBD found that Cambodia incurred 238,290 disability-adjusted life years (DALYs) in 1990, which increased to 306,245 DALYs in 2019. In 1990, Laos, Malaysia, and Myanmar incurred 29,232, 139,703, and 947,801 disability-adjusted life years (DALYs), respectively. In 2019, these countries experienced an increase in DALYs, with Laos, Malaysia, and Myanmar reporting 46,364, 182,303, and 1,448,635 DALYs, respectively. In 1990, the quantity of disability-adjusted life years (DALYs) recorded in Thailand was 676,741, which subsequently rose to 794,748 by 2019. The incidence of disability-adjusted life years (DALYs) in Vietnam increased from 1,117,224 in 1990 to 1,658,316 in 2019. Within the designated temporal scope, a significant number of years of life lost (YLLs) in mainland Southeast Asia were attributed to chronic respiratory diseases, including but not limited to chronic obstructive pulmonary disease and asthma, among other respiratory ailments. In 1990, Cambodia documented approximately 80,051 years of life lost (YLLs), an amount that rose to 101,328 YLLs in 2019. The YLLs in Laos were 10,271 in 1990 and increased to 15,156 in 2019. The YLLs in Malaysia were 42,112 in 1990 and increased to 51,715 in 2019. The number of years of life lost (YLLs) in Myanmar experienced an increase from 318,056 in 1990 to 492,813 in 2019. In 1990, Thailand experienced 272,644 years of life lost (YLLs), which subsequently rose to 328,846 in 2019. Likewise, it can be observed that in Vietnam, the years of life lost (YLLs) amounted to 323,631 in 1990 and exhibited an increase to 455,831 by 2019. Within the designated temporal parameters, chronic respiratory ailments exerted a substantial influence on the overall health of the populace residing in mainland Southeast Asia, leading to a noteworthy quantity of fatalities as well as a significant burden of disability-adjusted life years (DALYs) and years of life lost (YLLs). This highlights the necessity for continuous endeavors to alleviate air pollution and other hazardous elements linked with these ailments in this region.

**Figure 6:**
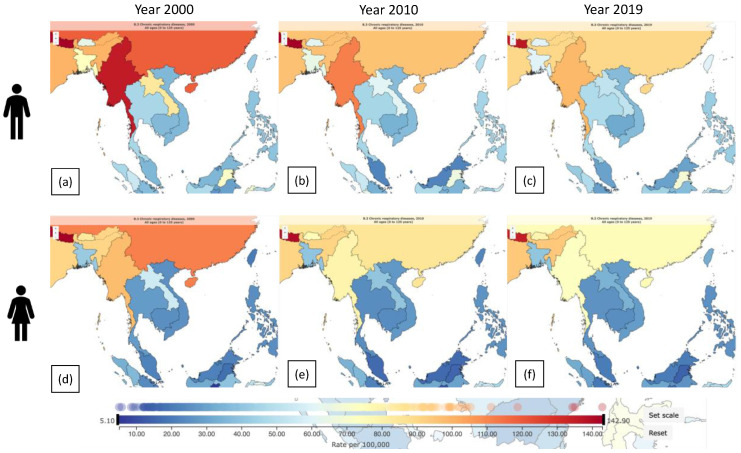
Deaths from chronic respiratory disease in Southeast Asia for males in (**a**) 2000, (**b**) 2010, and (**c**) 2019, and for females in (**d**) 2000, (**e**) 2010, and (**f**) 2019. (https://www.healthdata.org/gbd, accessed on 16 June 2023).

The mortality count arising from chronic respiratory ailments, namely chronic obstructive pulmonary disease, asthma, and other respiratory diseases, in Southeast Asia was found to display gender-based variations, as per the results of the Global Burden of Disease Study (GBD) 2000 [42] (Figure 6). The displayed fatality count for males and females within the specified region during 2000 was ascertained. In the context of Cambodia, the number of male deaths was recorded at 1661, while the number of female deaths was documented at 1530. In contrast, Laos reported significantly lower numbers, with 229 deaths among males and 199 among females. The recorded deaths in Malaysia were 3217 among males and 2457 among females. Based on the data, there were 13,487 reported deaths among males and 10,586 reported deaths among females in Myanmar. The recorded number of deaths in Thailand was 13,051 for males and 9638 for females. The mortality rate for males in Vietnam was 16,262, while for females, it was 13,491. According to available data, the number of fatalities in Cambodia in 2010 was approximately 2405 for males and 2208 for females. In Laos, the recorded count of male fatalities was 300, while the count of female fatalities was 259. The nation of Malaysia documented 4486 male deaths and 3318 female deaths. According to available data, the number of male fatalities in Myanmar was 20,222, whereas the number of female fatalities was recorded as 16,093. The number of recorded deaths for males in Thailand was 15,384, whereas, for females, it was 11,348. The number of fatalities documented in Vietnam was 21,477 for males and 17,624 for females. The evaluation of mortality rates for males and females in the region during the year 2019 is presently underway. According to the mortality statistics of Cambodia, there were 4327 documented deaths among males and 3958 documented deaths among females. In Laos, the number of recorded deaths among males was 448, while the number of recorded deaths among females was 387. The recorded deaths in Malaysia were 6491 for males and 4851 for females. From the currently available data, the mortality rate in Myanmar reveals that there were 30,569 deaths among males and 24,429 deaths among females. The recorded deaths in the Philippines were 33,448 among males and 27,390 among females. The recorded number of deaths among males in Thailand was 23,284, whereas the recorded number of deaths among females was 16,903. The recorded deaths in Vietnam were 34,286 among males and 28,175 among females. In the study by Baptista et al. [43], PM_2.5_ is strongly positively linked with chronic respiratory illness mortality, implying that increased particulate matter exposure increases mortality. Many Asian countries have experienced increased air pollution from industry and motor vehicle emissions in the recent decade [44]. Pollution is also caused by crop waste burning and bushfires in different Asian countries [45,46]. Furthermore, there was a rise in the incidence of asthma exposure to air pollution in the group of studies conducted on Asian populations investigated in the US-based Health Effects Institute (HEI) report [47], with a risk score of >1 and 2, enhancing a possibility for air contaminants to drive up the incidence of asthma [44].

Chongsuvivatwong et al. [48] previously examined the viewpoint regarding environmental and health issues in Southeast Asia. Emmanuel [49] reported a significant correlation between an elevation in the concentration of particulate matter (PM_10_) from 50 μg/m^3^ to 150 μg/m^3^ and a rise of 12% in upper respiratory tract illness, 19% in asthma, and 26% in rhinitis from public outpatient care facilities in Singapore. According to Mott et al. [50], the results of survival analyses indicated a statistically significant increase in the likelihood of readmission during the 1997 haze episode for individuals aged 65 years and older who had a history of hospitalization due to cardiorespiratory and respiratory diseases. A recent study has reported that the air quality index (AQI) is expected to increase to extremely unhealthy and hazardous levels during the dry season in the northern peninsula of Southeast Asia in the near future [6]. The elevated levels of PM_2.5_ pose a potential health hazard to people of all ages, especially children [6]. Infants are at a greater risk than other age groups, including toddlers, young children, school-age children, and adolescents [7]. Although adolescents face a lower risk of exposure to PM_2.5_, they still maintain a significant level of risk [7].

## 5. Air Quality Mitigation in Southeast Asia

The evidence suggests that air pollution is an issue that extends beyond the confines of a particular region [51]. The transboundary of air pollution necessitates the resolution of air pollution issues through either collaborative effort among regions or the establishment of worldwide environmental regulations, which are currently nonexistent [52]. There is an urgent requirement to develop transnational environmental laws that target the nations accountable for emitting pollutants and aim to hinder further environmental deterioration. It is possible for conscientious community members to address cross-border air pollution concerns collaboratively and amicably. Comprehending the significance of transboundary air pollution is a crucial aspect, as the outcomes emanating from diverse geographical origins furnish valuable insights for policymaking [53]. Understanding both local pollution and transboundary air pollution is crucial for contribution apportionment. Simultaneously, Southeast Asia exhibits a death of data from various nations within the vicinity, whereas a significant portion of the sedimentary archives that are accessible are inadequately resolved and insufficiently dated or lack any chronological information [54]. In the immediate future, it is imperative for Southeast Asian nations to contemplate enhanced collection and surveillance of pollution data. This can be achieved by augmenting the number of ground-based air quality observation stations, organizing regional field campaigns to differentiate air pollution in Southeast Asia, and instituting routine country-level Environmental Impact Assessments (EIAs) [53,54]. Comprehending the impact of regional pollution holds significance in the context of cross-border air pollution concerns. The acquisition of data pertaining to pollution in the vicinity has the capacity to furnish an impartial comprehension of the extent of pollution in areas that receive it. Furthermore, Luong [55] proposed several strategies to address the aforementioned concerns. These include the reduction of open burning of agricultural and municipal waste, the exploration of alternative options for the burning of agricultural waste, the enhancement of supervisory capacity for open biomass burning and associated air pollution, and the promotion of regional cooperation to combat open biomass burning and associated air pollution. Addressing this challenge necessitates that governments in the area undertake to endure financial and political obligations towards pollution monitoring and research and exhibit a willingness to exchange information and react efficiently to indications of chronically inadequate air quality. The provision of such information holds significant potential for environmental managers and policymakers operating at local, national, and regional levels.

In order to enhance the efficacy of air quality mitigation efforts in Southeast Asia, it is imperative that future scientific research be conducted and communicated to policymakers. As per the discourse presented by Chen and Taylor [54], the primary obstacle faced by Southeast Asia pertains to the dearth of data from a number of countries within the region. Furthermore, a significant proportion of the sedimentary records at hand are inadequately resolved, lack sufficient dating, or remain undated altogether. In the immediate future, it is imperative for Southeast Asian nations to prioritize the enhancement of pollution data collection and monitoring mechanisms. This can be achieved by increasing the number of ground-based air quality observation stations and organizing regional field campaigns that enable the identification of transboundary air pollution in the Southeast Asian region. Comprehending the impact of regional pollution is a crucial aspect of addressing transboundary air pollution concerns. The acquisition of data regarding pollution in the vicinity can furnish an impartial comprehension of pollution magnitudes in the receiving regions. An additional obstacle pertains to comprehending the conduct of pollutants during transportation through the utilization of chemical transport models (CTMs). In regions characterized by tropical climates and elevated levels of UV radiation and temperature, chemical alterations may occur during the transportation of these substances. Computational transport models (CTMs) have the capability to replicate alterations in levels of pollutants throughout the entirety of the transportation process. The modeling of secondary pollutants and microphysical alterations of aerosols poses significant challenges, particularly in Southeast Asia, where the region is primarily marine but still contains relatively dense forested areas. Accurately simulating the formation of secondary organic aerosol (SOA) in chemical transport models (CTMs) presents a challenge due to the coexistence of biogenic and anthropogenic SOA precursors, such as sea salt, reactive nitrogen, CO, and hydrocarbons. Challenges in achieving precise simulation of conditions can also emerge due to inadequate availability of precise emission inventory input data and a limited understanding of chemical conversions of contaminants in the surroundings. The outcomes of the model are subject to the estimated injection heights of smoke plumes, which have a direct impact on the simulation of pollutant mixing, chemical reactions, and transport distances. Accurately estimating the height of smoke injection in Southeast Asia poses a challenge owing to the diverse range of biomass burning practices and varying ambient meteorological conditions.

## 6. Conclusions

The issue of air pollution, specifically caused by particulate matter, has emerged as a significant concern across the Southeast Asian region in recent decades. The combustion of biomass is widely recognized to exert a substantial influence on the problem of air pollution in this region. The impact on individuals in this geographical area is substantial. Biomass burning in mainland Southeast Asia has been associated with the emergence of fires that pose a significant risk to air quality and are prevalent during the dry season. Over the past decades, the mortality rates of individuals related to air quality problems have exhibited a noteworthy increase. The estimated mortality rates linked to chronic respiratory diseases have detected a significant rise across most nations over the past decades. In the near future, air quality problems tend to increase the risk to human health in this region. The aforementioned findings underscore the persistent prevalence of chronic respiratory illnesses that are likely related to air quality problems in Southeast Asia and underscore the necessity of efficacious public health interventions aimed at mitigating the underlying risk factors that precipitate these fatalities.

## Data Availability

All data generated or analysed during this study are included in this published article.
